# Mucosal inverted closure of a post-gastric endoscopic submucosal dissection defect using grasping forceps with good rotatability and sharp claws

**DOI:** 10.1055/a-2466-9648

**Published:** 2024-12-03

**Authors:** Kaho Nakatani, Noriko Nishiyama, Kazuhiro Kozuka, Yukiko Koyama, Takanori Matsui, Tatsuo Yachida, Hideki Kobara

**Affiliations:** 1 38078 Gastroenterology and Neurology, Kagawa University Faculty of Medicine Graduate School of Medicine, Kita-gun, Japan

Although post-gastric endoscopic submucosal dissection (ESD) bleeding is reduced by defect closure 1 2 3 , there is no convenient and secure mucosal inverted closure method that enables early wound healing through sustained closure. We previously reported on post-ESD closure using jumbo grasping forceps (FG-47L-1; Olympus, Tokyo, Japan) 4 ; however, one problem was the poor maneuverability of the grasping forceps. Subsequently, we used EndoGrip grasping forceps (EndoGrip, AG-5039-2323; AGS MedTech, Tokyo, Japan) ( Fig. 1 ), which allows closure while inverting the mucosa. EndoGrip has two advantages: 1) small, sharp teeth at the tip and sharp claws in the arms that enable secure fold-and-drag maneuvers; and 2) good rotatability that provides easy maneuverability. We introduce a novel closure technique using EndoGrip forceps and endoclips.

**Fig. 1:**
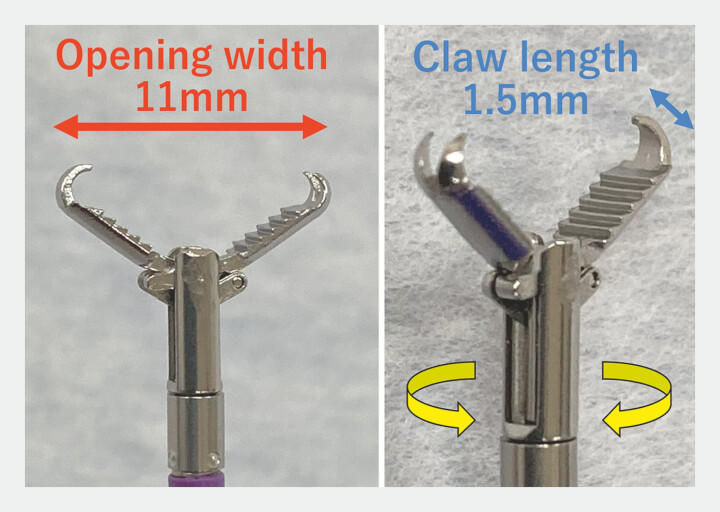
Photographs of the EndoGrip grasping forceps (AG-5039-2323; AGS MedTech, Tokyo, Japan), which has small, sharp teeth at the tip and sharp claws in the arms that enable secure fold-and-drag maneuvers, as well as having good rotatability that provides easy maneuverability; the claw length is 1.5 mm, with an opening width of 8.3 mm.

A 68-year-old man who was taking aspirin presented with a large early gastric cancer located on the lesser curvature in the angle. After standard ESD had been performed, a 38-mm defect remained ( Fig. 2 **a** ). After written informed consent had been obtained, the defect was closed using the following steps ( Fig. 3 ; Video 1 ). The EndoGrip was inserted into an endoscope with dual working channels (GIF-2TQ260M, Olympus), and one edge of the mucosal defect was grasped ( Fig. 2 **b** ). The grasped edge was dragged to the opposite edge of the mucosal defect, the EndoGrip was reopened, and the other side of the mucosa was grasped ( Fig. 2 **c** ). An endoclip (EZ Clip, HX-610-090L; Olympus) was inserted into the second channel of the endoscope, and the clip was pressed against the mucosa and closed while pulling the EndoGrip ( Fig. 2 **d,e** ). This procedure was repeated until the defect was completely closed ( Fig. 2 **f** ). Further endoclips were added in any gaps. The closure time was 31 minutes, and sustained closure was confirmed on postoperative days 3 and 7 ( Fig. 4 ).

**Fig. 2:**
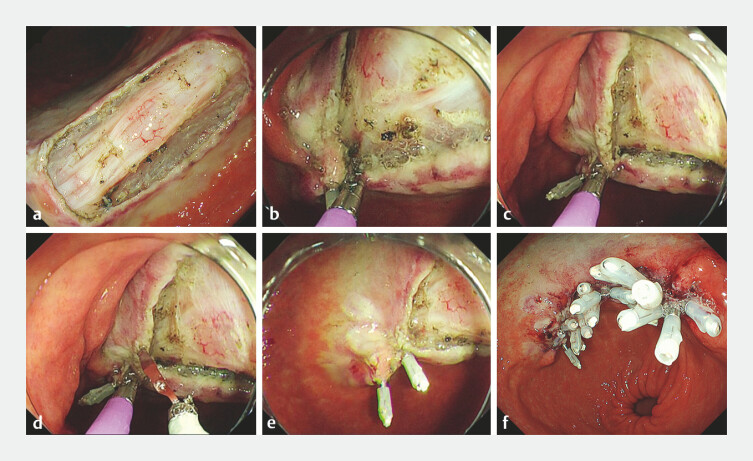
Endoscopic images of the closure procedure showing: **a** a 38-mm defect after standard endoscopic submucosal dissection; **b** the grasping forceps that had been inserted through one channel of a dual-channel endoscope being used to grasp one edge of the mucosal defect; **c** the grasped edge being dragged to the opposite edge of the mucosal defect, where the forceps is slowly reopened to grasp the other side of the mucosa; **d** an endoclip that had been inserted through the second channel of the endoscope being pressed against the mucosa and closed while pulling on the forceps; **e** the first two endoclips in place as the procedure is repeated along the entire defect length; **f** the completely closed defect.

**Fig. 3:**
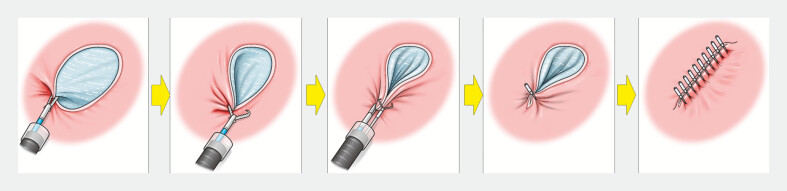
Schema of the procedure. Source: Davinch Medical Illustration Office.

**Fig. 4:**
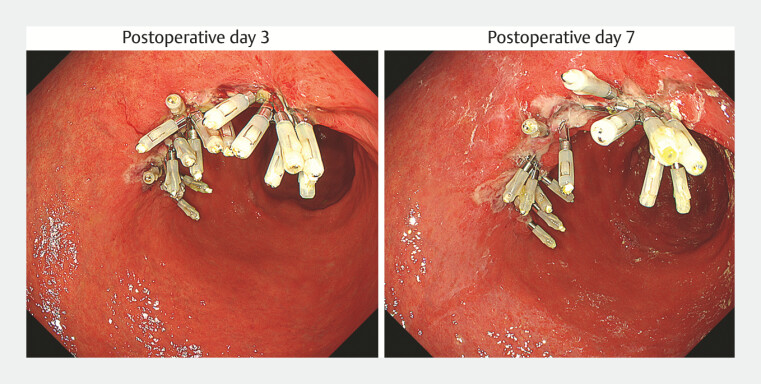
Endoscopic appearance on the 3rd and 7th postoperative days showing continued sustained closure, with all clips remaining in place.

Video showing the EndoGrip forceps being used to close an artificial gastric defect. Source for graphical illustrations: Davinch Medical Illustration Office.Video 1

The ease of maneuverability and high grasping strength of the EndoGrip simplify the technique of mucosal inverted gastric closure.

Endoscopy_UCTN_Code_TTT_1AO_2AO

## Notes

Endoscopy E-Videos https://eref.thieme.de/e-videos
*E-Videos* is an open access online section of the journal *Endoscopy* , reporting on interesting cases and new techniques in gastroenterological endoscopy. All papers include a high-quality video and are published with a Creative Commons CC-BY license. Endoscopy E-Videos qualify for HINARI discounts and waivers and eligibility is automatically checked during the submission process. We grant 100% waivers to articles whose corresponding authors are based in Group A countries and 50% waivers to those who are based in Group B countries as classified by Research4Life (see: https://www.research4life.org/access/eligibility/ ). This section has its own submission website at https://mc.manuscriptcentral.com/e-videos .
